# Supplementary material for: Newborn and child-like molecular signatures in older adults stem from TCR shifts across human lifespan
Source: Nat Immunol. 2023 Sep 25;24(11):1890–907. doi: 10.1038/s41590-023-01633-8 (PMC10602853; doi:10.1038/s41590-023-01633-8)
Supplement: Supplementary file 1 — Supplementary Table 1 [file 41590_2023_1633_MOESM1_ESM.pdf]

nature immunology

Article https://doi.org/10.1038/s41590-023-01633-8

Newborn and child-like molecular signatures in older adults stem from TCR shifts across human lifespan

In the format provided by the authors and unedited

Supplementary Table 1 Lifespan Cohort Demographics

Donor ID	Age group	Age (years)	Year of Birth	Sex	HLA-A	HLA-B	HLA-C	CMV status	TAME	Ex vivo TCR	Proliferation	scRNASeq
CB30	Newborn	0	2014	Unk	02:01 24:02	35:03 50:01	06:02 12:03	N.D.	Yes			
CB40	Newborn	0	2014	Unk	01:01 02:01	41:01 51:01	02:02 07:01	N.D.	Yes[c]			
CB59	Newborn	0	2015	F	01:01 02:01	08:01 41:01	02:08 07:01	N.D.	Yes			
CB60	Newborn	0	2015	M	01:01 02:01	08:01 41:01	02:08 07:01	N.D.	Yes			
CB140	Newborn	0	2018	Unk	02:01 32:01	44:02 51:01	02:02 05:01	Pos[a]	Yes			
CB143	Newborn	0	2018	Unk	02:01 03:01	07:02 15:01	03:03 07:02	Neg	Yes	Yes		Yes
CB146	Newborn	0	2018	Unk	02:01 68:02	14:02 51:01	01:02 08:02	Pos[a]	Yes	Yes		Yes
CB147	Newborn	0	2018	M	02:01 11:01	14:01 35:01	04:01 08:02	Neg	Yes	Yes		
CB149	Newborn	0	2018	M	02:01 03:01	07:02	07:02	Pos[a]	Yes	Yes	Yes	Yes
CB151	Newborn	0	2018	F	02:01 33:03	07:02 58:01	03:02 07:02	Pos[a]	Yes	Yes	Yes	
CB153	Newborn	0	2018	F	02:01	44:02	05:01 07:04	Neg	Yes	Yes	Yes	
TN012	Children	3	2016	M	02:01 29:02	44:03 51:01	14:02 16:01	Neg	Yes	Yes		
TN014	Children	5	2013	F	01:01 02:01	08:01 40:01	03:03 07:01	Pos	Yes	Yes		
TN024	Children	6	2012	M	02:01 32:03	15:01 44:02	04:01 05:01	Neg	Yes	Yes		Yes
TN004	Children	7	2011	M	02:01	44:02	05:01 07:04	Pos[b]	Yes	Yes		
TN022	Children	7	2011	F	01:01 02:01	07:02 37:01	06:02 07:02	Pos	Yes	Yes	Yes	
TN010	Children	8	2011	F	02:01	08:01 15:01	04:01 07:01	Neg	Yes	Yes		
TN001	Children	9	2010	F	01:01 02:01	08:01 44:02	05:01 07:01	Neg	Yes	Yes		
TN003	Children	9	2009	M	02:01 29:02	44:02 44:03	05:01 16:01	Pos	Yes	Yes	Yes	
TN029	Children	12	2007	M	02:01 02:05	49:01	07:01	Neg	Yes	Yes		Yes
F3058C56	Children	14	1998	F	02:01	41:02 44:02	05:01 17:03	N.D.	Yes	Yes		Yes
TN007	Children	14	2004	F	02:01 03:01	14:02 44:02	05:01 08:02	Pos	Yes	Yes	Yes	
TN016	Children	16	2002	F	02:01 11:01	15:01 40:01	03:03 03:04	Neg	Yes	Yes		
BP141	Adults	18	2001	M	02:01 03:01	07:02 38:01	07:02 12:03	Neg	Yes	Yes		
KK116	Adults	24	1995	F	02:01 03:01	15:01 44:02	03:03 05:01	Neg	Yes			
BP145	Adults	24	1995	F	02:01	27:05 44:03	01:02 16:01	Neg	Yes			
KK86	Adults	26	1992	M	02:01 11:01	51:01 55:01	02:02 03:03	Neg	Yes			Yes
BP128	Adults	29	1989	F	02:01 30:01	44:02 58:01	05:01 07:18	N.D.	Yes	Yes	Yes	
KK83	Adults	29	1988	F	02:01	15:22 18:01	07:01 12:03	Pos	Yes			
KK2	Adults	33	1985	F	02:01 11:01	35:01 39:01	Unk	Neg	Yes	Yes	Yes	Yes
BP152	Adults	34	1985	M	02:01 26:01	38:01 55:01	03:02 12:03	N.D.	Yes			
BP154	Adults	36	1982	M	01:01 02:01	07:02 37:01	06:02 07:02	Neg	Yes			
KK11	Adults	36	1982	M	02:01 11:01	35:01 39:01	Unk	Pos	Yes	Yes		
BP129	Adults	37	1981	F	01:01 02:01	07:02 57:01	06:02 07:02	N.D.	Yes	Yes	Yes	
KK42	Adults	37	1981	M	01:01 02:01	07:02 44:02	05:01 44:02	Neg	Yes	Yes	Yes	Yes
KK18	Adults	48	1970	F	02:01 03:02	18:01 35:08	Unk	Pos	Yes	Yes		
BP131	Adults	50	1968	F	02:01 03:01	15:01 47:01	03:03 06:02	N.D.	Yes			
BP42	Adults	51	1961	Unk	02:01 11:01	07:02 44:02	Unk	N.D.	Yes			
BP86	Adults	51	1965	M	02:01	07:02 39:01	07:02 13:03	N.D.	Yes	Yes		
BP117	Adults	57	1961	M	02:01 03:01	07:02 40:01	03:04 07:02	N.D.	Yes			
BP134	Adults	57	1961	F	02:01 32:01	35:03 40:02	04:01 15:02	N.D.	Yes			
BP157	Adults	57	1962	M	01:01 02:01	18:01 35:01	04:01 07:01	Pos	Yes			
BP87	Adults	58	1958	M	02:01 32:01	40:01 44:02	03:04 05:01	N.D.	Yes			
DMC31	Older Adult	63	1954	M	02:01 03:01	07:02 50:01	06:02 07:02	Pos	Yes	Yes		
BP164	Older Adult	65	1954	Unk	02:01 30:01	13:01 44:03	06:02 16:01	Pos	Yes			
DMC54	Older Adult	65	1954	F	02:01	15:01 27:05	03:04 06:02	Pos	Yes			
BP45	Older Adult	67	1945	Unk	01:01 02:01	13:02 44:32	Unk	N.D.	Yes	Yes		
BP127	Older Adult	68	1950	F	02:01 30:01	18:01 44:02	05:01 07:01	N.D.	Yes	Yes	Yes	
BP163	Older Adult	68	1951	Unk	01:01 02:01	08:01 14:01	07:01 08:02	Pos	Yes			
DMC12	Older Adult	70	1948	F	02:01 24:02	27:05 40:01	Unk	Neg	Yes[c]			
DMC18	Older Adult	71	1947	F	02:01	15:01 44:02	Unk	Pos	Yes	Yes	Yes	Yes
BP149	Older Adult	72	1947	M	02:01 03:01	35:01 40:02	02:02 04:01	Pos	Yes			
BP162	Older Adult	72	1946	Unk	02:01 11:01	07:02 44:02	05:01 07:02	Pos	Yes			

BP114	Older Adult	73	1945	F	02:01	11:01	40:01		03:04		N.D.	Yes	Yes	Yes	
DMC51	Older Adult	74	1944	F	02:01	24:02	35:03	44:03	04:01	12:03	Neg	Yes	Yes		
DMC47	Older Adult	76	1943	F	01:01	02:01	07:02	08:01	07:01	07:02	Pos	Yes	Yes		
DMC42	Older Adult	77	1941	F	02:01	03:01	15:01	35:03	03:04	12:03	Pos	Yes			
DMC3	Older Adult	79	1939	F	02:01		07:02	44:02	05:01	07:02	Pos	Yes	Yes	Yes	
DMC48	Older Adult	79	1939	F	01:01	02:01	08:01	14:02	07:01	08:02	Pos	Yes	Yes		Yes
DMC20	Older Adult	86	1931	M	02:01	03:01	27:05	44:02	Unk		Pos	Yes[c]			
DMC19	Older Adult	88	1930	F	01:01	02:01	08:01	13:02	Unk		Pos	Yes	Yes		Yes

N.D=not determined, no plasma available, Pos=CMV positive, Neg=CMV negative, [a] most likely maternal antibodie, [b] Equivocal

[c] Total of <10 counted A2/M1$_{58-66}$$^{+}$CD8^{+} T cells within whole enrich fraction, sufficient for analysis of frequencies but not phenotypes
