# Supplementary material for: Newborn and child-like molecular signatures in older adults stem from TCR shifts across human lifespan
Source: Nat Immunol. 2023 Sep 25;24(11):1890–907. doi: 10.1038/s41590-023-01633-8 (PMC10602853; doi:10.1038/s41590-023-01633-8)
Supplement: Supplementary file 2 — Reporting Summary [file 41590_2023_1633_MOESM2_ESM.pdf]

nature portfolio

Corresponding author(s): Katherine Kedzierska

Last updated by author(s): Jul 31, 2023

Reporting Summary

Nature Portfolio wishes to improve the reproducibility of the work that we publish. This form provides structure for consistency and transparency in reporting. For further information on Nature Portfolio policies, see our Editorial Policies and the Editorial Policy Checklist.

Statistics

For all statistical analyses, confirm that the following items are present in the figure legend, table legend, main text, or Methods section.

n/a	Confirmed	
☐	☒	The exact sample size (*n*) for each experimental group/condition, given as a discrete number and unit of measurement
☐	☒	A statement on whether measurements were taken from distinct samples or whether the same sample was measured repeatedly
☐	☒	The statistical test(s) used AND whether they are one- or two-sided<br>*Only common tests should be described solely by name; describe more complex techniques in the Methods section.*
☒	☐	A description of all covariates tested
☐	☒	A description of any assumptions or corrections, such as tests of normality and adjustment for multiple comparisons
☐	☒	A full description of the statistical parameters including central tendency (e.g. means) or other basic estimates (e.g. regression coefficient) AND variation (e.g. standard deviation) or associated estimates of uncertainty (e.g. confidence intervals)
☐	☒	For null hypothesis testing, the test statistic (e.g. *F*, *t*, *r*) with confidence intervals, effect sizes, degrees of freedom and *P* value noted<br>*Give P values as exact values whenever suitable.*
☒	☐	For Bayesian analysis, information on the choice of priors and Markov chain Monte Carlo settings
☒	☐	For hierarchical and complex designs, identification of the appropriate level for tests and full reporting of outcomes
☒	☐	Estimates of effect sizes (e.g. Cohen's *d*, Pearson's *r*), indicating how they were calculated

Our web collection on statistics for biologists contains articles on many of the points above.

Software and code

Policy information about availability of computer code

Data collection	LSR Fortessa II (BD Biosciences), BD FACSAria III (BD Biosciences) ; NextSeq500 platform
Data analysis	FlowJo v10.8.1; Prism v9.3.0; R v.4.2.2, TCRdist3, Trimmomatic v0.39, TopHat v2.1.1, Cufflinks v2.2.1, VDJPuzzle v3, Seurat v4.1.0, MAST v1.16.0, Scanpy v1.7.1, FinchTV v1.5.0, MASS package, v7.3.58

For manuscripts utilizing custom algorithms or software that are central to the research but not yet described in published literature, software must be made available to editors and reviewers. We strongly encourage code deposition in a community repository (e.g. GitHub). See the Nature Portfolio guidelines for submitting code & software for further information.

Data

Policy information about availability of data

All manuscripts must include a data availability statement. This statement should provide the following information, where applicable:

- Accession codes, unique identifiers, or web links for publicly available datasets
- A description of any restrictions on data availability
- For clinical datasets or third party data, please ensure that the statement adheres to our policy

Data will be made available according to our Data availability statement provided in the manuscript
Data availability
TCR sequence data (ex vivo Supplementary Table 2; in vitro Supplementary Table 3, Source Data) will be deposited into VDJdb [https://vdjdb.cdr3.net] following manuscript acceptance. The published article includes all datasets generated or analyzed during the study in the source data file. scRNA-seq data that support the study is deposited in NCBI-GEO with accession GSE237817 and will be available following publication of the manuscript. This paper does not report original code. Any additional information required to reanalyze the data reported in this paper is available from the lead contact upon request.

Specific details:
TCR sequence data will be deposited into VDJdb [https://vdjdb.cdr3.net] following manuscript acceptance and will be linked to the PMID
scRNA-seq data that support the study will be deposited in NCBI-GEO with accession GSE237817 following manuscript acceptance.
A source data file will be generated to show all data points.
Raw FACS data are shown in the manuscript. FACS-source files are available upon request.
Msigdb was used to perform GSEA and was accessed automatically by the GSEA R package fgsea function.

Field-specific reporting

Please select the one below that is the best fit for your research. If you are not sure, read the appropriate sections before making your selection.

☒ Life sciences ☐ Behavioural & social sciences ☐ Ecological, evolutionary & environmental sciences

For a reference copy of the document with all sections, see nature.com/documents/nr-reporting-summary-flat.pdf

Life sciences study design

All studies must disclose on these points even when the disclosure is negative.

Sample size	The sample size was determined by the availability of samples.
Data exclusions	No data were excluded with the following exception: Donors who had a total number of less then 10 counted A2M1+CD8+ T cells within the whole enriched fraction were excluded for further phenotypic analysis as cell numbers were to low. This was indicated in the manuscript.
Replication	Experiments in figure 1-7 could not be replicated due to limited PBMC numbers. SKW-3 cell line experiments (figure 8) were repeated in 2 or 3 independent experiments as indicated in the figure legends.
Randomization	N/A. Donors were selected from a large randomly recruited and HLA-typed lifespan cohort n = >500, consisting of 154 newborns, 30 children, ~300 adults and 57 older adults, based on their expression of HLA-A*02:01. Samples were used based on availability of material.
Blinding	Experiments were not blinded, instead all analysis have been independently checked by multiple researches

Reporting for specific materials, systems and methods

We require information from authors about some types of materials, experimental systems and methods used in many studies. Here, indicate whether each material, system or method listed is relevant to your study. If you are not sure if a list item applies to your research, read the appropriate section before selecting a response.

Materials & experimental systems

n/a	Involved in the study	
☐	☒	Antibodies
☐	☒	Eukaryotic cell lines
☒	☐	Palaeontology and archaeology
☒	☐	Animals and other organisms
☐	☒	Human research participants
☒	☐	Clinical data
☒	☐	Dual use research of concern

Methods

n/a	Involved in the study	
☒	☐	ChIP-seq
☐	☒	Flow cytometry
☒	☐	MRI-based neuroimaging

Antibodies

Antibodies used

We used commercially-available antibodies as per Material and Methods.

TAME and proliferation
Surface staining: anti-CD71-BV421 (Clone M-A712, 1:50, BD Biosciences #562995, TAME only), anti-CD3-BV510 (Clone OKT3, 1:200, BioLegend #317332), anti-HLA-DR-BV605 (Clone L243, 1:100, BioLegend 307640), anti-CD4-BV650 (Clone SK3, 1:100, BD Biosciences #563875), anti-CD27-BV711 (Clone L128, 1:200, BD Horizon #563167), anti-CD38-BV785 (Clone HIT2, 1:100, BD Biosciences #563964), anti-CD57-APC (Clone NK-1, 1:400, BD Biosciences #560845), anti-CCR7-AF700 (Clone 150503, 1:50, BD Biosciences #561143), anti-CD14-APC-Cy7 (Clone MϕP91:100, BD Biosciences #560180), anti-CD19 (Clone SJ25C1, 1:100, BD Biosciences #560177), anti-CD45RA-FITC (Clone HI100, 1:200, BD Biosciences #555488), anti-CD8-PerCP-Cy5.5 (1:200, BD Biosciences #565310), anti-CD95-PECF594 (Clone SK1, 1:100, BD Horizon #562395), anti-PD1-1-PE-Cy7 (Clone EH12.1, 1:50, BD Biosciences #561272), Live/Dead fixable aqua dead-cell stain (1:800, Invitrogen #L10119), cell trace violet (Violet Proliferation Dye 450, BD Horizon, proliferation only)

ICS
Surface staining: anti-CD3-BV510 (Clone OKT3, 1:200, Biolegend #317332), anti-CD8-BV605 (Clone SK1, 1:200, BD Horizon #564116),

anti-CD4-BV650 (Clone SK3, 1:200, BD Horizon #563875), anti-CD27-BV711 (Clone L128, 1:200, BD #563167), anti-CD14-APC-H7 (Clone MϕP91:100, 1:100, BD Pharmingen #560180), anti-CD19-APC-H7 (Clone SJ25C1, 1:100, BD Pharmingen #560177), Live/Dead near-infrared (1:800, Invitrogen #L10119), anti-CD45RA-FITC (Clone HI100, 1:200, BD Biosciences #555488), anti-CD95-PECF594 (Clone SK1, 1:100, BD Horizon #562395).
Intracellular staining: anti-TNFa-APC (Clone 6401.1111, 1:100 BD, #340534), anti-Granzyme B-AF700 (Clone GB11, 1:50, BD #560213), anti-IFNy-FITC (Clone 4S.B3, 1:100, eBioscience #45-7-319-42), Perforin (Clone B-D48, 1:10, Biolegend #353316)

TCR cell lines
anti-CD3-BV421 (1:100, BD Biosciences #562426), Live/Dead fixable aqua dead-cell stain (1:500, Invitrogen #L10119), CD69-PE-Cy7 (Clone FN50, 1:100, BD #557745)

Validation

All antibodies were validated for use on human cells by the manufacturers, and were titrated in our laboratory before use.

Eukaryotic cell lines

Policy information about cell lines

Cell line source(s)

HEK293T cells were obtained from ATCC (www.atcc.org),
C1RA2 cells were obtained from the Chen laboratory (La Trobe University), C1R parental cell line was obtained from the Department of Biochemistry and Molecular Biology & Infection and Immunity Program, Biomedicine Discovery Institute Monash University
SKW-3-CD3 and SKW-3-CD3+CD8 were obtained from the McCluskey Laborartory (University of Melbourne)

Authentication

Cell lines were not formally authenticated. C1R-A2 cells were routinely tested for HLA expression level prior to each experiment. Presence of CD8 and/or CD3 in SKW-3 lines were confirmed by GFP expression

Mycoplasma contamination

Cell lines tested negative for mycoplasma contamination

Commonly misidentified lines
(See ICLAC register)

No commonly misidentified cell lines were used in this study

Human research participants

Policy information about studies involving human research participants

Population characteristics

Please refer to Supplementary Tables 1 for details

Recruitment

Samples were recruited through the University of Melbourne (UoM). Australian Red Cross Blood Service (ARCBS), Deepdene Medical Clinic (DMC) (through co-author J. Crowe), Launceston General Hospital (through co-author K. Flanagan), St Jude Children's Research Hospital (through co-author P. Thomas), Mercy Hospital for Women (through co-author M. Lappas). All donors were recruited randomly and on voluntary basis.
Signed informed consents were obtained from all blood donors or their guardians prior to the study. Participants of the study did not receive any compensation.

Ethics oversight

Experiments conformed to the Declaration of Helsinki Principles and the Australian National Health and Medical Research Council Code of Practice. Written informed consent was obtained from all blood donors or their guardians prior to the study. The study was approved by the Human Research Ethics Committee (HREC) of the University of Melbourne (Ethics ID #24567; #13344; #23852), Australian Red Cross Lifeblood (ID 2015#8), St Jude Children's Research Hospital (XPD12-089 IIBANK), Mercy Hospital for Women (#R14-25) and Tasmanian Health and Medical Human Research Ethics Committee (ID H0017479).

Note that full information on the approval of the study protocol must also be provided in the manuscript.

Flow Cytometry

Plots

Confirm that:

☒ The axis labels state the marker and fluorochrome used (e.g. CD4-FITC).

☒ The axis scales are clearly visible. Include numbers along axes only for bottom left plot of group (a 'group' is an analysis of identical markers).

☒ All plots are contour plots with outliers or pseudocolor plots.

☒ A numerical value for number of cells or percentage (with statistics) is provided.

Methodology

Sample preparation

Samples were prepared as described in Methods

Instrument

BD LSRII Fortessa and BD FACSAriaIII were used for acquisition of data, BD FACS AriaIII was used for single cell index sorting

Software

BD FACS Diva, FlowJo

Cell population abundance

Only single cell sorting was performed, which was confirmed by the presence of single TCR strains

Gating strategy

Gating strategy has been described in Figure 1c, Figure 7c and f, Figure 8b,c,e, g,and i, extended data figure 1a, extended data figure 8a,c and e and extended data figure 9a

TAME: FSC-A vs SSC-A gating --> doublet exclusion (FSC-A vs FSC-H) --> dead cell exclusion (LD staining). Immune cells were gated by classical markers (e.g. CD3, CD4 and CD8). T cell subsets were defined by CD27, CD45RA and CD95. A2/M1 specific cells were identified by teramer staining. Boolean gating was performed for CD57, PD1, CD38, HLADR and CD71 among viable CD8+ or tetramer+ populations

TAME: FSC-A vs SSC-A gating --> doublet exclusion (FSC-A vs FSC-H) --> dead cell exclusion (LD staining). Immune cells were gated by classical markers (e.g. CD3, CD4 and CD8). T cell subsets were defined by CD27, CD45RA and CD95. A2/M1 specific cells were identified by teramer staining. Boolean gating was performed for CD57, PD1, CD38, HLADR and CD71 among viable CD8+ or tetramer+ populations

Proliferation: similar to TAME, except for CD71 staining which was not included. Instead proliferating tetramer populations were identified by the loss of VPD450

ICS: FSC-A vs SSC-A gating --> doublet exclusion (FSC-A vs FSC-H) --> dead cell exclusion (LD staining). Immune cells were gated by classical markers (e.g. CD3, CD4 and CD8). T cell subsets were defined by CD27, CD45RA and CD95. A2/M1 specific cells were identified by teramer staining. Boolean gating was performed for IFNy, TNFa, GrzB and Perforin among viable CD8+ or tetramer+ populations. Proliferating tetramer populations were identified by the loss of VPD450

TCR cell lines: FSC-A vs SSC-A gating --> doublet exclusion (FSC-A vs FSC-H) --> dead cell exclusion (LD staining). TCR expressing cell lines were identified based on CD3 and GFP coexpression. A2/M1 specificity was established by teramer staining on CD3+GFP+ cells. T cell activation was measured by CD69 expression on GFP positive cells.

☒ Tick this box to confirm that a figure exemplifying the gating strategy is provided in the Supplementary Information.
